# High‐Efficiency Non‐Fullerene Acceptors Developed by Machine Learning and Quantum Chemistry

**DOI:** 10.1002/advs.202104742

**Published:** 2022-01-06

**Authors:** Qi Zhang, Yu Jie Zheng, Wenbo Sun, Zeping Ou, Omololu Odunmbaku, Meng Li, Shanshan Chen, Yongli Zhou, Jing Li, Bo Qin, Kuan Sun

**Affiliations:** ^1^ MOE Key Laboratory of Low‐Grade Energy Utilization Technologies and Systems School of Energy and Power Engineering Chongqing University 174 Shazhengjie Shapingba Chongqing 400044 China; ^2^ Bremen Center for Computational Materials Science University of Bremen Am Fallturm 1 Bremen 28359 Germany; ^3^ College of Chemistry and Chemical Engineering Chongqing University Chongqing 400044 China

**Keywords:** density functional theory (DFT), electrostatic potential (ESP), machine learning, non‐fullerene acceptors, organic photovoltaics

## Abstract

Y6 and its derivatives have greatly improved the power conversion efficiency (PCE) of organic photovoltaics (OPVs). Further developing high‐performance Y6 derivative acceptor materials through the relationship between the chemical structures and properties of these materials will help accelerate the development of OPV. Here, machine learning and quantum chemistry are used to understand the structure–property relationships and develop new OPV acceptor materials. By encoding the molecules with an improved one‐hot code, the trained machine learning model shows good predictive performance, and 22 new acceptors with predicted PCE values greater than 17% within the virtual chemical space are screened out. Trends associated with the discovered high‐performing molecules suggest that Y6 derivatives with medium‐length side chains have higher performance. Further quantum chemistry calculations reveal that the end acceptor units mainly affect the frontier molecular orbital energy levels and the electrostatic potential on molecular surface, which in turn influence the performance of OPV devices. A series of promising Y6 derivative candidates is screened out and a rational design guide for developing high‐performance OPV acceptors is provided. The approach in this work can be extended to other material systems for rapid materials discovery and can provide a framework for designing novel and promising OPV materials.

## Graphical abstract

Twenty‐two new Y6 derivatives acceptor materials with the predicted power conversion efficiency higher than 17% are screened out by using machine learning. Combing the trends discovered by machine learning and quantum chemistry calculations, it is further found out the ways in which side chains and end acceptor units affect the performance of OPV.

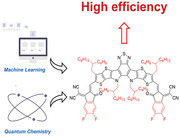

## 1 Introduction

Organic photovoltaic (OPV) cells based on non‐fullerene acceptor materials have made great progress in recent years and are expected to achieve highly efficient power conversion as the form of renewable energy. Since the star acceptor material Y6 was developed,^[^ ^1^ ^]^ the power conversion efficiency (PCE) of OPV has been improved continuously, and now it has exceeded 18%.^[^ ^2^, ^3^ ^]^ However, the conventional procedure for experimenters to develop new acceptors is by synthesizing molecules with various possible structures and then test their photoelectric conversion performance, which is time and resource consuming. Therefore, screening molecules in a chemical space (all possible molecular structures) in advance to obtain potential OPV molecules with high performance will save resources and accelerate the development of new OPV materials.

Due to the complexity of organic photovoltaic cells, it is difficult to establish a direct relationship between the chemical structure of materials and the performance of OPV devices using traditional computational simulation methods. In recent years, with the development of artificial intelligence, researchers have tried to use machine learning^[^ ^4^ ^]^ to establish the aforementioned structure–performance relationship. As a data‐driven method, machine learning can learn from existing data, build relationships, and make predictions, even without relevant background knowledge.^[^ ^5^ ^]^

Machine learning has been widely used to predict the PCE of OPV devices and screen new OPV materials.^[^ ^6^, ^7^, ^8^ ^]^ Recently, some outstanding works related to feature engineering^[^ ^9^, ^10^ ^]^ and machine learning algorithms^[^ ^11^, ^12^ ^]^ have been reported to improve the prediction accuracy. Interestingly, some have gone further and tried to obtain structure characteristics of high‐performance OPV materials by machine learning models. For example, Sahu et al.^[^ ^13^ ^]^ extracted the effect of unit changes on the photoelectric conversion performance through machine learning by using molecular descriptors computed from quantum chemistry as input and then dividing the predicted OPV material molecules into multiple functional units. Wu et al.^[^ ^14^ ^]^ used one‐hot encoding to convert OPV donor–acceptor pairs into input, and then they successfully obtained some high PCE donor–acceptor pairs by combing machine learning and experiments. However, few works can provide the design principles of new high‐performance OPV materials for experimentalists. And the existing works are based on old databases which lack new Y6 series acceptors. Although there are thousands of OPV experimental data, they are almost useless for the new material system of Y6. It is challenging to build the structure–performance relationship for Y6 series acceptors and discover new high‐efficiency Y6 derivative acceptors due to the small database available.

In this work, we established an OPV database with Y6 and its derivatives as acceptor materials. The acceptor molecules were divided into three parts and encoded by an improved one‐hot code as input for machine learning. The machine learning model based on Random Forest^[^ ^15^ ^]^ (RF) algorithm shows a good predictive ability and was used to screen the chemical space formed by all possible molecular structures. Our machine learning model screened out 22 new high‐potential OPV acceptor materials with a predicted efficiency greater than 17%. The screening results show that high‐performance OPV non‐fullerene derivatives of Y6 acceptors typically possess medium‐length side chains. Further quantum chemistry calculations show that the end acceptor unit of molecules mainly modifies the frontier molecular orbital energy levels and the electrostatic potential (ESP) at the molecular surface, thereby leading to differences in photoelectric conversion performance.

## 2 Results

**Figure 1:**
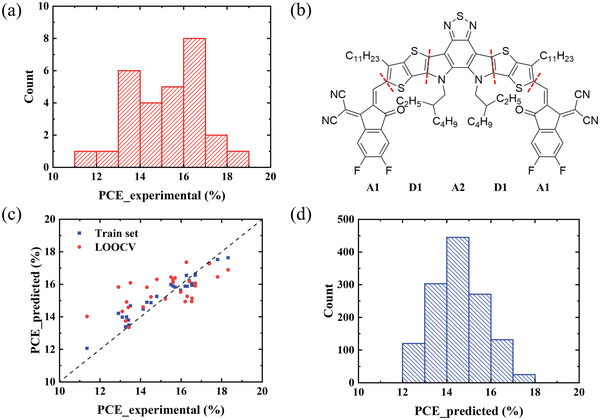
a) Statistical distribution of measured PCEs in the database. b) Acceptor molecules splitting method. c) Machine learning model evaluation showing good fit for the training set (blue) and the cross‐validation test set (red), via the leave‐one‐out cross‐validation (LOOCV) method. d) The predicted PCE of OPV devices in the new virtual database.

At first, we collected the performance data of all organic solar cells employing Y6 and its derivatives as acceptor materials from literature. To investigate the relationship between acceptor molecular structures and OPV devices performance, the donor materials were limited to PBDB‐T or PBDB‐TF (PM6). The chemical structures of the acceptor materials along with their corresponding device PCE were then recorded to establish the original database. The PCE of OPV devices distribution in the database is shown in **Figure** **1**a. The PCE of all the 29 OPV devices in the database are over 10%, and the maximum PCE value is as high as 18.32%.^[^ ^3^ ^]^

In order to facilitate the learning of the relationship between molecular structure and device performance, it is necessary to convert the molecules into code that can be recognized by computers.^[^ ^12^ ^]^ Since all these acceptor molecules are symmetrical A‐D‐A‐D‐A type^[^ ^1^ ^]^ molecules, as shown in Figure 1b, we can split the molecules into three parts, i.e., the end acceptor unit (A1), the donor unit (D1), and the core acceptor unit (A2). These fragmented units were then encoded with an improved one‐hot encoding approach. As shown in **Table** **1** and Table S1 (Supporting Information), unique bits are used to encode various fragments of the acceptor molecules. The one‐hot encoding of molecular fragments was performed such that if two fragments differ only in side chain length, they are regarded as the same type of fragment and encoded according to the length of the side chain (e.g., the A2 fragments of both molecules shown in Table 1). The code of the acceptor materials is obtained by splicing the code of three units. We use simple integers—0 and 1, to encode the two possible donor materials employed in this study. Finally, the code of donor material and acceptor material are spliced to obtain an input for the machine learning model.

**Table 1:** Fragments coding method of OPV acceptor materials

	A1	D1	A2
Molecule1	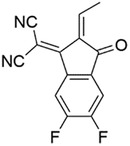	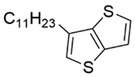	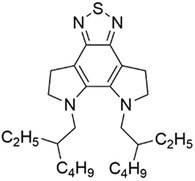
Code1	0 1 0 0 0 0	4 0 0 0 0 0	1 0 0 0 0
Molecule2	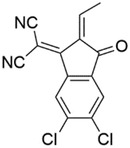	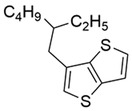	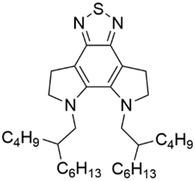
Code2	0 0 1 0 0 0	0 1 0 0 0 0	2 0 0 0 0

According to previous research, ensemble methods, such as RF and Gradient Boosted Decision Tree^[^ ^16^ ^]^ (GBDT), are suitable for the fingerprint like input.^[^ ^11^, ^14^ ^]^ We chose the RF algorithm to learn the relationship between the input that contains the molecular structures and the PCE of their based OPV devices. Figure 1c reports the fitting results of the machine learning model on the training set and the performance of the machine learning model when using leave‐one‐out cross‐validation (LOOCV). LOOCV take all the samples except one as training set and the sample left out as test set. This means that all data in the original database will be tested and no random factors affect the evaluation data, thereby making model evaluation more reliable.^[^ ^4^ ^]^ The mean absolute error (MAE) and correlation coefficient *r* on the training set are 0.43 and 0.97, respectively. While the MAE and *r* on the validation set are 1.08 and 0.66, respectively. The performance of the machine learning model on the training set is excellent, implying that the algorithm achieved a good fit to the input data. The performance on the validation set is worse than that on the training set because part of the fragment has only appeared once in the database. When the molecules with these fragments are divided into the validation set, machine learning cannot make reasonable prediction for the unlearned features. Nevertheless, the performance of the machine learning model on the training set and validation set is good enough to expect that the machine learning model can make accurate predictions for the new combinations based on the fragments as all of them have been learned by machine learning algorithm.

In order to traverse all possibilities, all A1‐D1‐A2 permutations of the molecule fragments in the original database were paired with both donor materials to generate a new virtual database. This virtual database contains 1296 possible donor–acceptor combinations and the machine learning model trained above is used to predict the PCE value of their corresponding organic solar cells. The predicted PCEs of the OPV devices in the new virtual database is shown in Figure 1d. More than 10% of the combinations have predicted PCE greater than 16% and there are 25 combinations with the PCE greater than 17%. All the donor molecules are PM6 and the chemical structures of these 25 acceptor molecules are shown in Table S2 (Supporting Information). Among these 25 acceptor molecules, there are only 3 molecules in the original database, the predicted PCE and experimental PCE of which show the same change trend. The other 23 acceptor molecules are completely new and it turns out that these high‐performance acceptor molecules predicted by machine learning have similar structures and they only differ in side chains and the end acceptor units.

**Figure 2:**
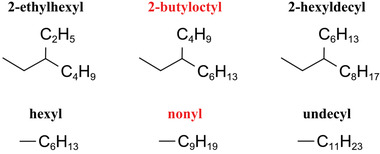
The structure of the side chains.

Considering side chains, 19 of the best performing molecules possess 2‐butyloctyl as the side chain. A drop in predicted PCE is usually observed when 2‐butyloctyl is replaced. When the A1 and D1 units are identical, the predicted PCE of molecules with 2‐butyloctyl as the side chain on the A2 unit is usually the highest (e.g., C1 higher than C2, C3). On D1 unit, 2‐butyloctyl usually shows higher performance than 2‐ethylhexyl and 2‐hexyldecyl (e.g., C1 higher than C6, the other lower than 17%), while nonyl usually shows higher PCE than hexyl and undecyl (e.g., C4 higher than C12, the other lower than 17%). The structure of these side chains is shown in **Figure** **2**. In general, our model predicts that molecules with medium‐length side chains exhibit higher photoelectric conversion performance. This observation may as a result of the realization of balance between solubility and crystallinity.^[^ ^3^, ^17^ ^]^

**Figure 3:**
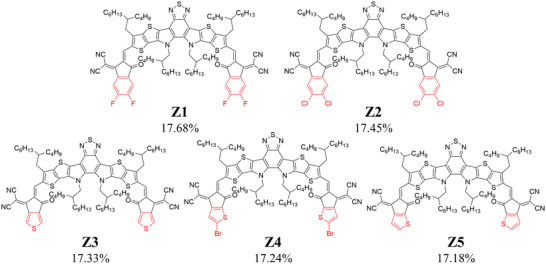
Five typical machine learning predicted high‐performance acceptor molecules with different acceptor units at the end groups (highlighted in red) and their predicted PCE.

For the A1 unit, the machine learning model provides a prediction but the general trend with changing A1 units is unclear. To further understand the impact of end acceptor unit, we selected 5 typical molecules denoted by Z1, Z2, Z3, Z4, and Z5, respectively. Their chemical structures and predicted PCE are shown in **Figure** **3**. The only difference between these molecules is the acceptor unit at the ends and the predicted PCE of Z1 and Z2 are higher than that of Z3, Z4 and Z5. Quantum chemical calculations were performed to further analyze the effect of end groups on the molecular properties.

**Figure 4:**
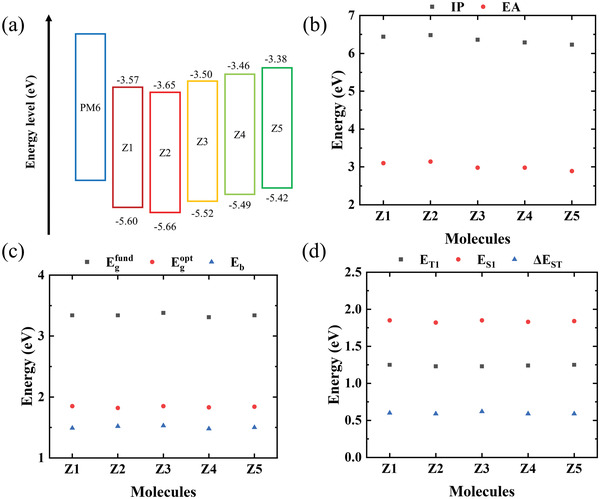
Electronic properties of Z1–Z5 molecules. a) Frontier molecular orbital energy levels in comparison with the PM6 donor molecule. b) Ionization potentials (IP, marked as gray squares) and electron affinities (EA, marked as red circles). c) Fundamental gaps (*E*_g_^fund^_,_ marked as gray squares), optical gaps (*E*_g_^opt^, marked as red circles), and electron–hole pair binding energies (*E*_b_, marked as blue triangles). d) The lowest singlet excitation energies (*E*_S1_, marked as red circles), lowest triplet excitation energies (*E*_T1_, marked as gray squares), and singlet–triplet energy gaps (Δ*E*_ST_, marked as blue triangles).

Firstly, we calculated the frontier molecular orbital energy levels of these five molecules. The proper energy level alignment between the molecular orbitals of the donor and acceptor materials is very important in photoelectric conversion. The differences in highest occupied molecular orbital (HOMO) energy levels and lowest unoccupied molecular orbital (LUMO) energy levels between donors and acceptors influence exciton dissociation.^[^ ^18^ ^]^ Moreover, the difference between LUMO energy level of acceptor and HOMO energy level of donor is directly related to the open circuit voltage of the OPV device.^[^ ^19^ ^]^ Although the energy levels measured by cyclic voltammetry are not the same as the molecular orbital energy levels calculated by quantum chemistry methods,^[^ ^20^ ^]^ it has been shown that they exhibit the same trend to a certain extent. As shown in **Figure** **4**a, the HOMO‐LUMO gaps of the five molecules are approximately the same. The HOMO energy level and LUMO energy level of molecule Z2 are slightly lower than that of molecule Z1, while the front orbital energy levels of molecules Z3, Z4, Z5 are higher than those of molecule Z1 and Z2. In general, with the exception of Z2, the HOMO and LUMO energy levels of the molecules increase with the order of their numbering. The ionization potential (IP) and electron affinity (EA) of these five molecules shown in Figure 4b show the same change trend as the frontier molecular orbital energy levels. Combined with their predicted PCE values from the machine learning model, the frontier orbital energy levels of the Z1 and Z2 molecules seem to provide a better match with the PM6 donor material.

The difference between fundamental gap (*E*_g_^fund^) and optical gap (*E*_g_^opt^) is the electron–hole pair binding energy (*E*_b_).^[^ ^20^ ^]^ The smaller the *E*_b_, the easier it is for electron and hole to dissociate.^[^ ^21^ ^]^ The calculated results shown in Figure 4c indicate that the electron–hole pair binding energy of the five molecules are very close, and they are all relatively small due to the excellent performance of the main structure derived from Y6. The difference between the lowest singlet excitation energy (*E*_S1_) and lowest triplet excitation energy (*E*_T1_) is the singlet–triplet energy gap (Δ*E*_ST_), and a lower Δ*E*_ST_ is conducive to simultaneously realize an enhancement in light absorption and a reduction of energy losses.^[^ ^22^, ^23^ ^]^ The calculation results of TD‐DFT shown in Figure 4d indicate that the Δ*E*_ST_ of the five molecules are also close, indicating that they withal yield high performance devices. This is in excellent agreement with the predicted high PCE values from the machine learning model.

**Figure 5:**
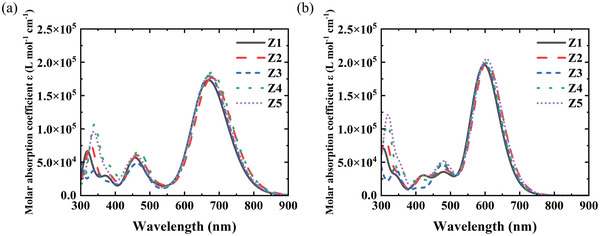
a,b) UV–vis absorption spectra of the five molecules calculated by B3LYP and tuned *ω* parameter of long range corrected *ω*B97XD functionals, respectively.

The UV–vis absorption spectra of these five molecules calculated using TD‐DFT with two different functionals are shown in **Figure** **5**. Except for shifts in absorption peak positions due to their different Hartree–Fock components, the shapes and relative intensities of the optical absorption peaks are almost the same. The absorption profile of all five molecules in the long wavelength region are quite similar, and their maximum absorption peak positions only differ slightly. On the contrary, their absorption spectra vary significantly in the short wavelength. The absorption intensity of molecule Z4 and Z5 in the short‐wave region are higher than the others, initially indicating superior photon absorption yield from the incident solar spectrum. However, considering that the absorption of the donor material and the acceptor material are complementary (shown in Figure S1, Supporting Information), the absorption in the short‐wave region has a limited impact on the overall photovoltaic performance.

**Figure 6:**
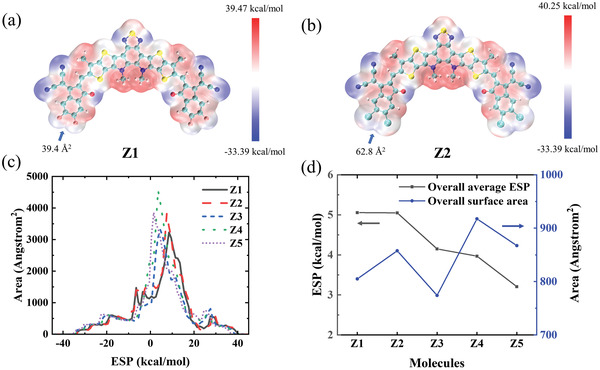
The ESP distribution on molecular surface of molecule a) Z1 and b) Z2. c) Statistics of the ESP distribution on molecular surface. d) The overall average ESP on molecular surface and overall surface area of five molecules.

Recently, the electrostatic potential (ESP) distribution on the molecular surface of OPV materials has attracted a lot of attention^[^ ^24^, ^25^ ^]^ due to its influence on molecular packing. In addition, the ESP difference between donor and acceptor molecules provides a driving force for exciton dissociation. The ESP, *V* (*r*), at the surface of a molecular is defined as sum of the Coulomb potential of each nucleus and electrons^[^ ^26^ ^]^

(1) $$V\left(r\right)=\sum_{A}\frac{Z_{A}}{\left|R_{A}-r\right|}-\int \frac{\rho \left(r^{\prime }\right)}{\left|r^{\prime }-r\right|}\;dr^{\prime }$$

where *V* (*r*) is written in terms of atomic units. *Z*_A_ is the charge on nucleus *A*, *R*_A_ is the coordinates of *A*, and *ρ* (*r*) is the charge density. Computations of ESP at the van der Waals surface of these five molecules were performed according to the method outlined in ref. [27] and the results are shown in **Figure** **6** and Figure S2 (Supporting Information).

The ESP distribution on these five molecular surfaces is similar, with significant dissimilarities occurring near the end acceptor units. As shown in Figure 6a,b, surface area of the end unit in molecule Z2 is larger than that of Z1, due to a larger radius of Cl atom compared to the F atom. The larger positive ESP area of Z2 facilitates exciton dissociation. Figure 6c shows the statistics of molecular surface ESP. It turns out that molecule Z1 and Z2 show more distribution in areas of high ESP than other molecules, which indicating that Z1 and Z2 can generate a stronger driving force for exciton dissociation when the acceptor interacts with the donor to form an intermolecular electric field. The larger overall average ESP of Z1 and Z2 compared to the other molecules (shown in Figure 6d) furthermore agrees with the observation that molecules Z1 and Z2 possess a stronger ability for driving exciton dissociation while interacting with the donor material. Since Z2 takes lager overall surface area than Z1, it shows greater possibilities to interact with the donor due to the larger area of positive ESP. In addition, although the average ESP of Z3 is slightly larger than that of Z4, the overall surface area of Z3 is much less than that of Z4. Combining with statistics of the ESP distribution (Figure 6c), Z4 shows higher potential than Z3.

## 3 Conclusion

In summary, we trained a machine learning model based on the OPV database constructed from Y6 and its derivatives. The machine learning model shows good predictive ability, and 22 new acceptor materials with predicted PCE higher than 17% were screened from the new virtual database. The acceptor molecules predicted to have higher performance possess medium‐length side chains. Quantum chemistry calculations on five high performance molecules with the same donor unit but with different acceptor units reveal that these molecules mainly show differences in frontier molecular orbital energy levels and the ESP distribution on the molecular surface. These differences were shown to be the origin in their different photoelectric properties. Our work has screened out a series of OPV acceptor materials with high potential, and provided a rational design guide for the development of high‐performance OPV materials. The approach in this work, for investigation of the relationship between the molecular structure of OPV materials and the PCE of their based OPV devices, can be extended to other material systems for rapid materials discovery and can provide a rational framework for the design of novel and promising OPV materials.

## 4 Experimental Section

### Machine Learning Algorithm and Model Evaluation

Random forest (RF) algorithm was used to establish the structure–performance relationship between OPV materials and PCE of their based OPV devices. The number of trees in the forest was set to 50. The small size of the data set required that leave‐one‐out cross‐validation (LOOCV) technique was used for cross‐validation of the training set, for reliable model evaluation. Mean Absolute Error (MAE) and correlation coefficient *r* were employed to evaluate the machine learning model.

(2) $$\mathrm{MAE}=\frac{1}{n}\sum \left|x_{\mathrm{predicted}}-x_{\mathrm{experimental}}\right|$$

(3) $$r=\frac{Cov\left(x_{\mathrm{predicted}},x_{\mathrm{experimental}}\right)}{\sqrt{Var\left(x_{\mathrm{predicted}}\right)Var\left(x_{\mathrm{experimental}}\right)}}\;$$

where *Cov* is the covariance and *Var* is variance of data.

The machine learning algorithms, model evaluation, and other related statistical analysis were performed using scikit‐learn^[^ ^28^ ^]^ library on Python 3.x.

### Quantum Chemistry Calculations

Density functional theory (DFT) and time‐dependent density functional theory (TD‐DFT) were used to calculate the geometric structures and properties of molecules. The side chains of all molecules were removed in these calculations as they are identical. Since OPV usually worked in the form of solid films and the solvents were sometimes different even when tested in solvent environment, the solvent mode was not considered.

Geometry optimization and vibration analysis were performed at B3LYP/6‐31G(d,p) level^[^ ^29^, ^30^ ^]^ combined with DFT‐D3 (BJ) dispersion correction.^[^ ^31^, ^32^ ^]^ The optimized structures were obtained at the minimum energy points and with no imaginary frequencies.

The frontier orbital energy levels, vertical ionization energies, and excited states of the molecules were calculated using B3LYP functional combined with 6‐31G(d,p) basis set. The vertical electron affinity was calculated by combining the 6‐31+G(d,p) basis set.^[^ ^33^ ^]^ The UV–vis absorption spectra of the molecules were obtained by calculating the first 80 excited states and broadening by Gaussian function. The full width at half maximum (FWHM) was set to 0.33333 eV.

All DFT and TD‐DFT calculations were performed by Gaussian program.^[^ ^34^ ^]^ The analysis of wavefunction was performed by Multiwfn^[^ ^35^ ^]^ and visualization was performed by VMD.^[^ ^36^ ^]^

HOMO‐LUMO gap (*E*_g_^HM^) was equal to the difference between LUMO and HOMO energy levels.

(4) $$E_{g}^{\mathrm{HM}}=E\left(\mathrm{LUMO}\right)-E\left(\mathrm{HOMO}\right)$$

where *E* is the electronic energy of the system.

Ionization potential (IP) is defined as:

(5) $$\mathrm{IP}=E\left(n-1\right)-E\left(n\right)$$

Electron affinity (EA) is defined as:

(6) $$\mathrm{EA}=E\left(n\right)-E\left(n+1\right)$$

where *n* is the original number of electrons.

Fundamental gap (*E*_g_^fund^) is equal to the difference between IP and EA.

(7) $$E_{g}^{\mathrm{fund}}=\mathrm{IP}-\mathrm{EA}$$

Electron–hole pair binding energy (*E*_b_) is equal to the difference between fundamental gap and optical gap.

(8) $$E_{b}=E_{g}^{\mathrm{fund}}\;-E_{g}^{\mathrm{opt}}$$

Singlet–triplet gap (Δ*E*_ST_) is equal to the difference between the lowest singlet excitation energy (*E*_S1_) and the lowest triplet excitation energy (*E*_T1_)

(9) $$\Delta E_{\mathrm{ST}}=E_{S1}\;-E_{T1}$$

The above properties were all calculated iteratively via a tuned *ω* parameter of long range corrected functional *ω*B97XD^[^ ^37^ ^]^ and the iteration results are shown in the Supporting Information. The conclusions are consistent with those obtained by the B3LYP functional. The optimal *ω* parameter has a value of 0.0943, which was optimized by minimizing function *J* ^2^

(10) $$J^{2}\left(\omega \right)=J_{n}^{2}\;\left(\omega \right)+J_{n+1}^{2}\left(\omega \right)$$

where

(11) $$J_{n}^{2}\left(\omega \right)={\left[E^{\omega }\left(\mathrm{HOMO},\;n\right)+\mathrm{IP}\left(n\right)\right]}^{2}$$

(12) $$J_{n+1}^{2}\;\left(\omega \right)={\left[E^{\omega }\left(\mathrm{HOMO},\;n+1\right)+\mathrm{IP}\left(n+1\right)\right]}^{2}$$

## Conflict of Interest

The authors declare no conflict of interest.

## Supporting information

Supporting Information

## Data Availability

The data that support the findings of this study are available from the corresponding author upon reasonable request.
