# Supplementary material for: High‐Efficiency Non‐Fullerene Acceptors Developed by Machine Learning and Quantum Chemistry
Source: Adv Sci (Weinh). 2022 Jan 6;9(6):2104742. doi: 10.1002/advs.202104742 (PMC8867193; doi:10.1002/advs.202104742)
Supplement: Supplementary file 1 — Supporting Information [file ADVS-9-2104742-s001.pdf]

ADVANCED SCIENCE
Open Access

Supporting Information

for *Adv. Sci.,* DOI: 10.1002/advs.202104742

High-efficiency non-fullerene acceptors developed by machine learning and quantum chemistry

Qi Zhang, Yujie Zheng, Wenbo Sun, Zeping Ou, Omololu Odunmbaku, Meng Li, Shanshan Chen, Yongli Zhou, Jing Li, Bo Qin, Kuan Sun**

Supporting Information

High-efficiency non-fullerene acceptors developed by machine learning and quantum chemistry

Qi Zhang, Yujie Zheng, Wenbo Sun, Zeping Ou, Omololu Odunmbaku, Meng Li, Shanshan Chen, Yongli Zhou, Jing Li, Bo Qin, Kuan Sun**

This supporting information presents the following contents.

Table S1. Molecular fragments of accepter materials and their corresponding codes.

Table S2. Acceptor molecules with predicted PCE greater than 17% in the virtual database. The experimental PCE are in brackets.

Table S3. Frontier molecular orbital energy levels and HOMO-LUMO gaps calculated by the two functionals.

Table S4. Ionization potential (IP), electron affinity (EA) and fundamental gap (E_g^{fund}) calculated by the two functionals.

Table S5. The lowest singlet excitation energy (E_{S1}), lowest triplet excitation energy (E_{T1}) and singlet-triplet energy gap (ΔE_{ST}) calculated by the two functionals.

Table S6. The electron–hole pair binding energy (E_b) calculated by the two functionals.

Figure S1. UV-Vis absorption spectra of Z1 and PM6 calculated by B3LYP functional.

Figure S2. The ESP on the van der Waals surface of the five molecules.

References

Table S1. Molecular fragments of accepter materials and their corresponding codes.

<table>
<tr><th colspan="2">A1</th></tr>
<tr><td>100000</td><td>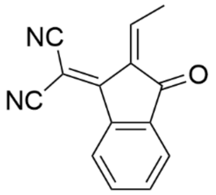
</td></tr>
<tr><td>010000</td><td>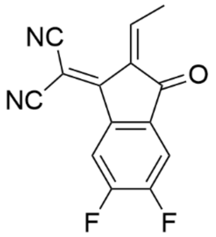
</td></tr>
<tr><td>001000</td><td>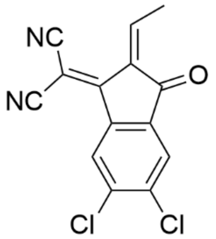
</td></tr>
<tr><td>000100</td><td>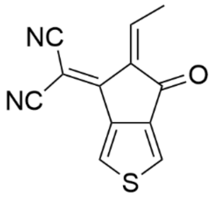
</td></tr>
<tr><td>000010</td><td>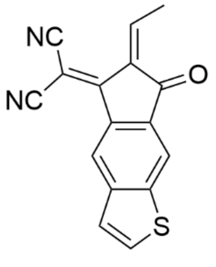
</td></tr>
<tr><td>000001</td><td>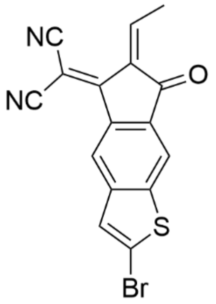
</td></tr>
<tr><td colspan="2"></td></tr>
</table>

D1	
100000	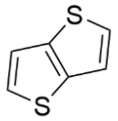
200000	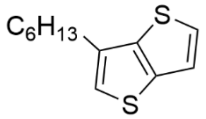
300000	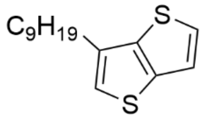
400000	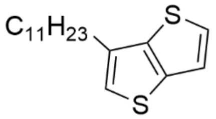
010000	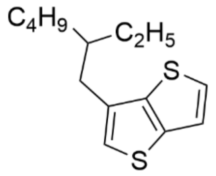
020000	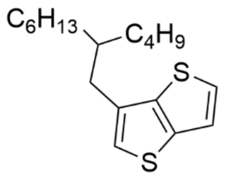
030000	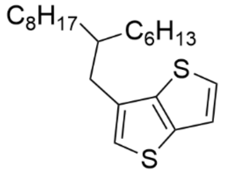
040000	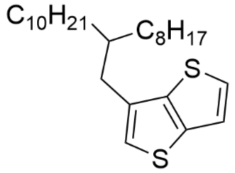
001000	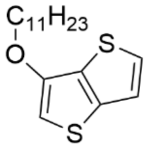
000100	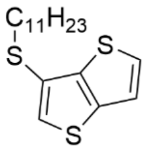

000010	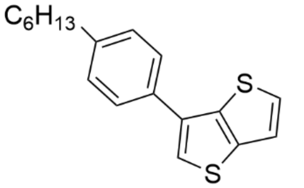
000001	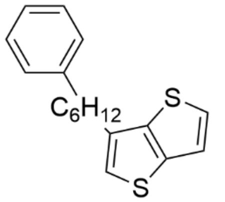
**A2**	
10000	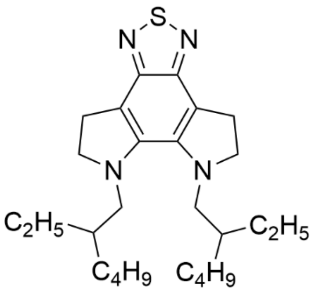
20000	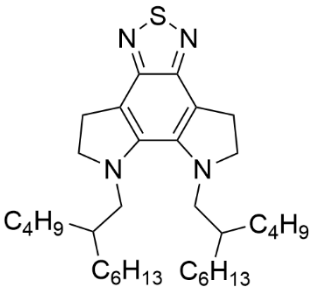
30000	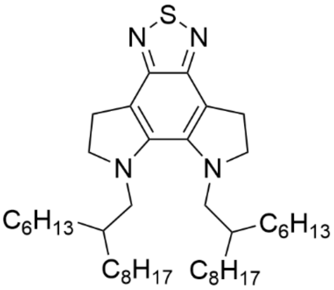

01000	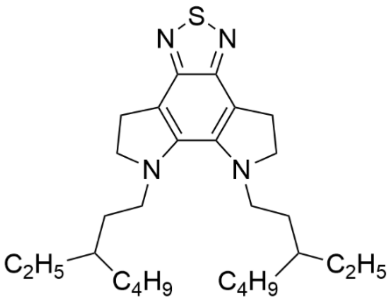
02000	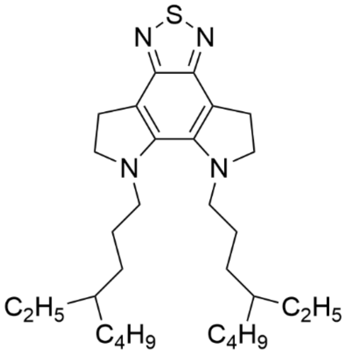
00100	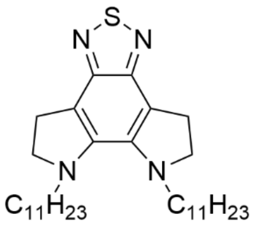
00200	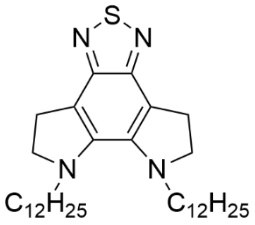
00010	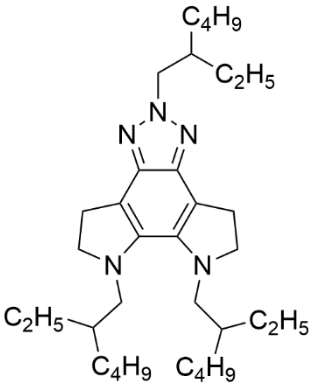

00001	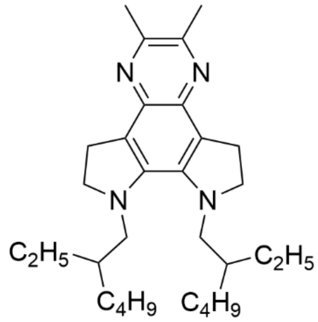

Table S2. Acceptor molecules with predicted PCE greater than 17% in the virtual database. The experimental PCE are in brackets.

number	PCE (%)	molecule
C1	17.68	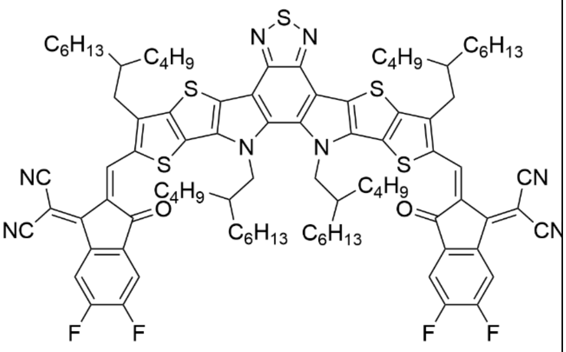
C2 (L8-BO[1])	17.64 (18.32)	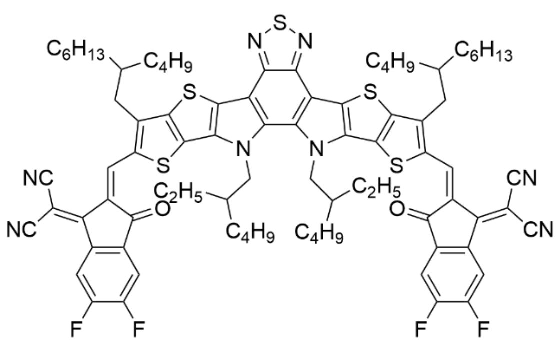
C3	17.59	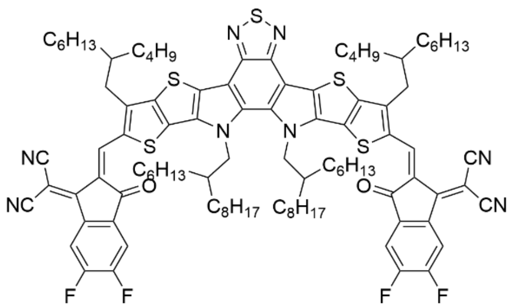

C4 (BTP-eC9[2])	17.52 (17.8)	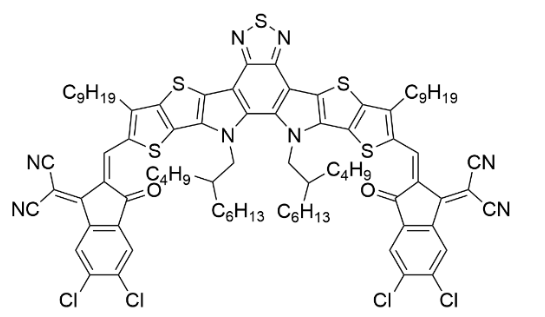
C5	17.45	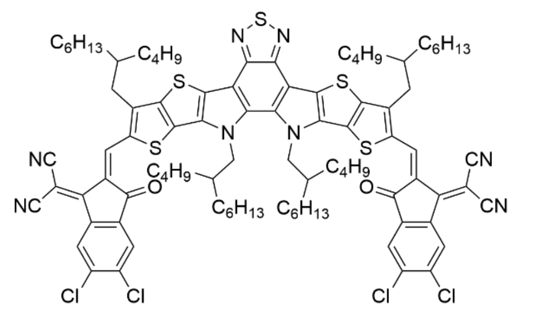
C6	17.36	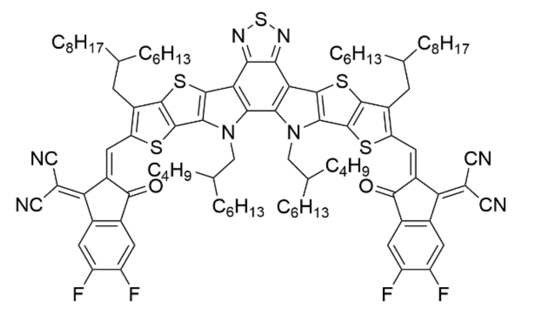
C7	17.34	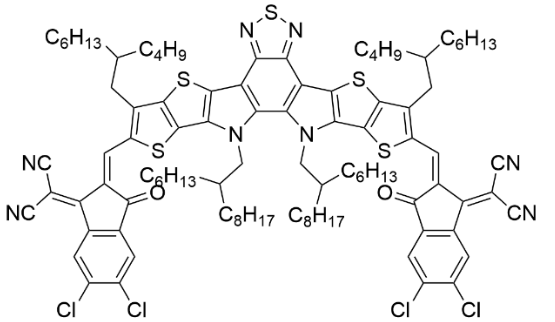
C8	17.33	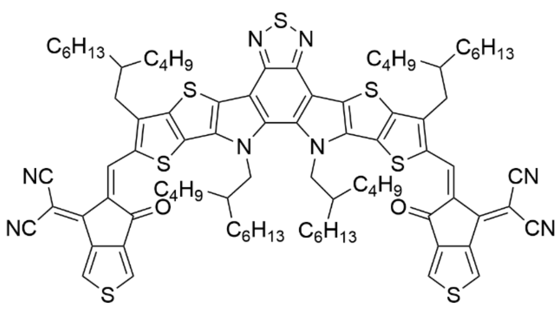

C9 (L8-HD[1])	17.31 (17.39)	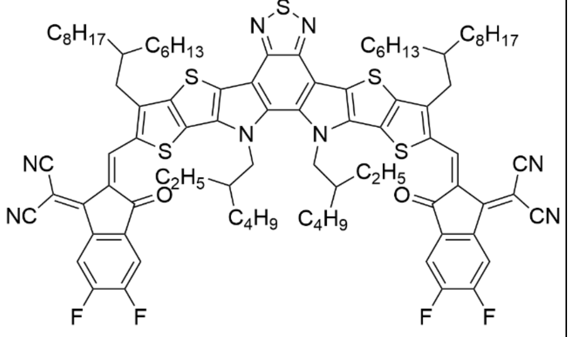
C10	17.27	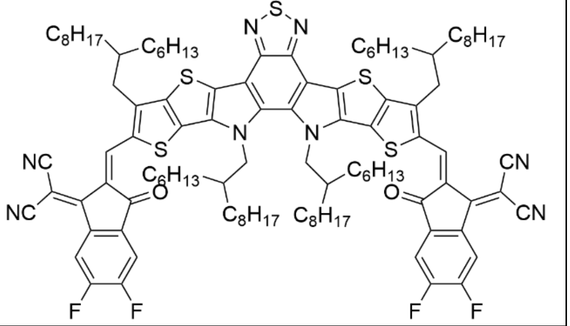
C11	17.24	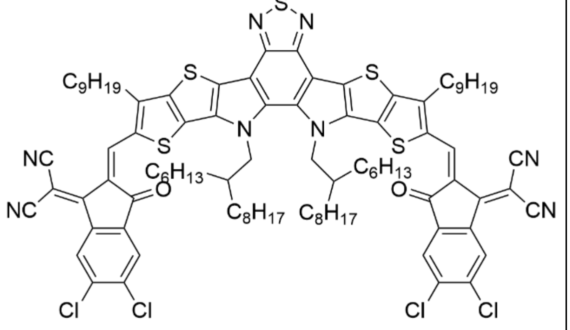
C12	17.24	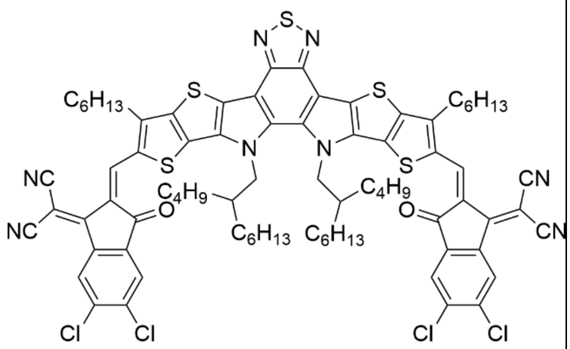
C13	17.24	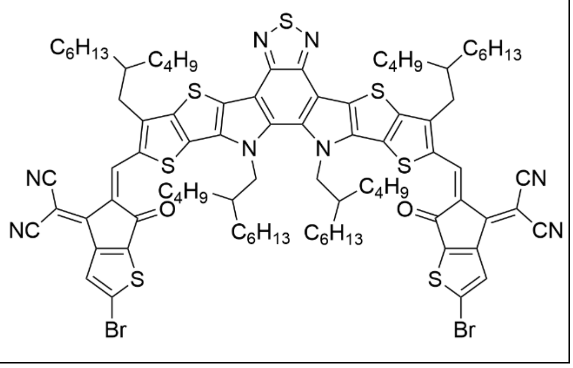

C14	17.23	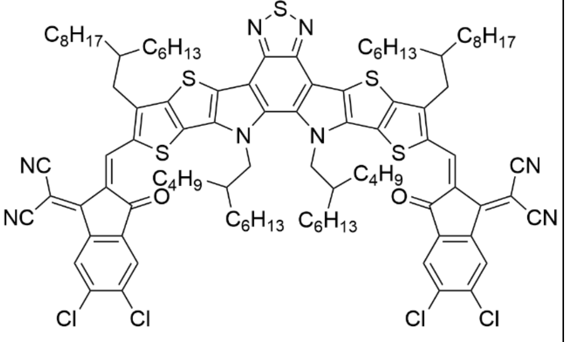
C15	17.22	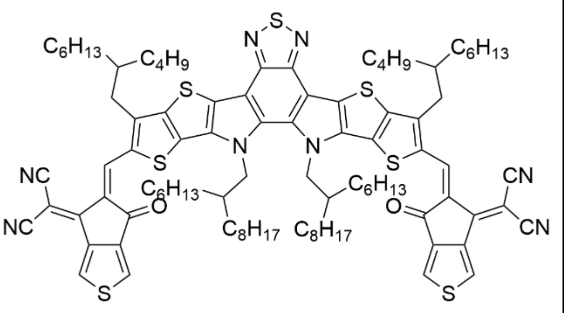
C16	17.18	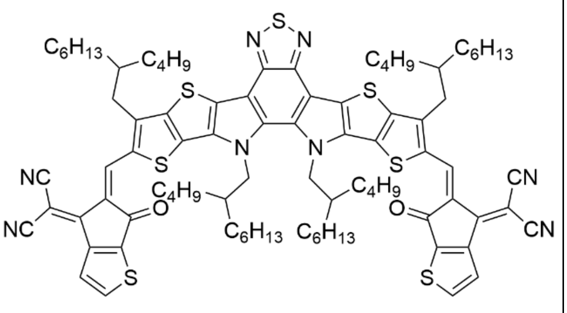
C17	17.13	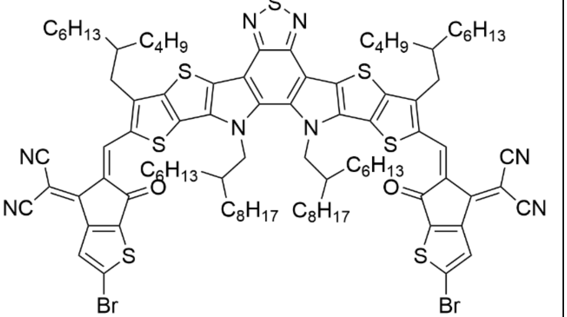
C18	17.12	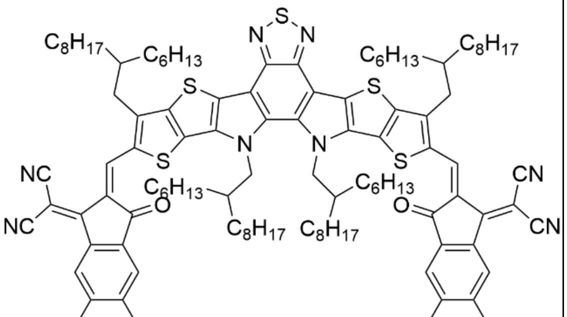

C19	17.12	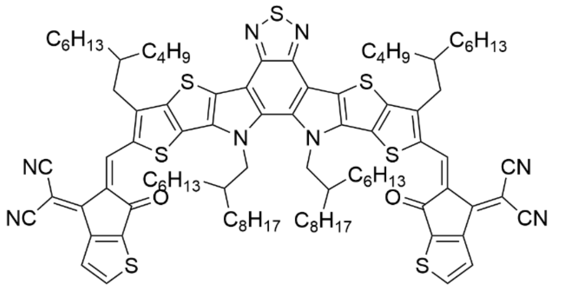
C20	17.08	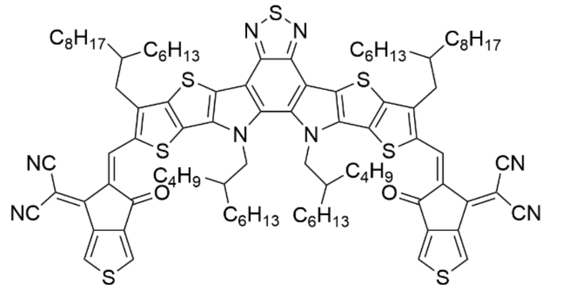
C21	17.04	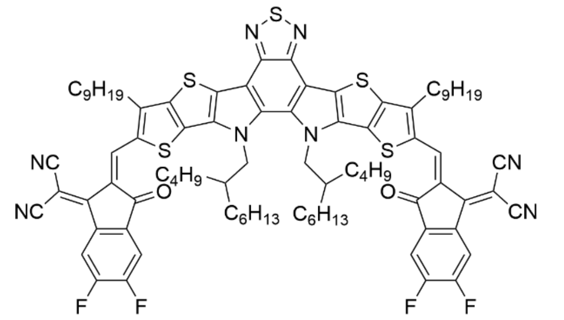
C22	17.02	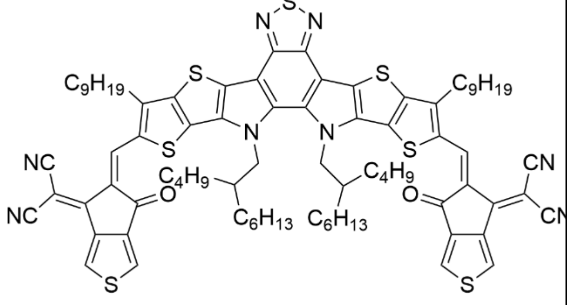
C23	17.02	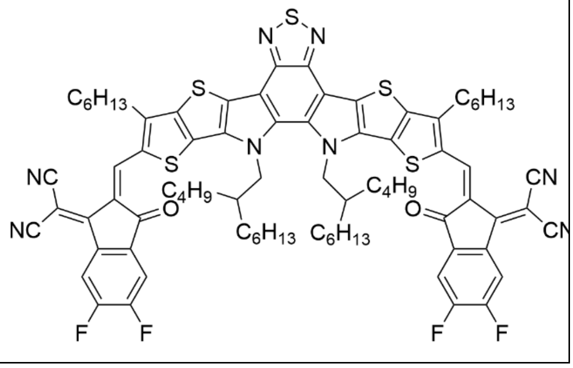

C24	17.02	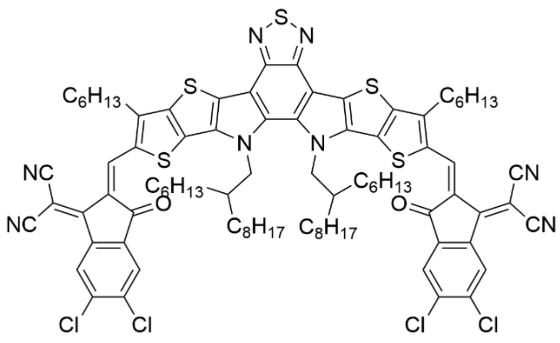
C25	17.00	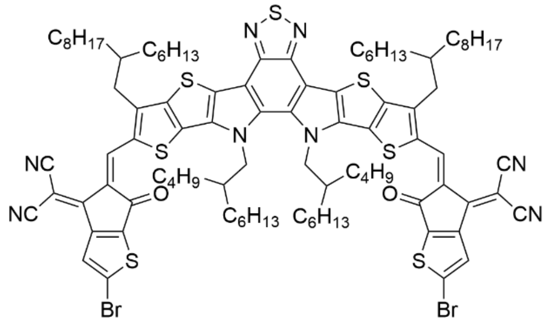

Table S3. Frontier molecular orbital energy levels and HOMO-LUMO gaps calculated by the two functionals.

	B3LYP-D3			ωB97XD		
	HOMO/eV	LUMO/eV	E_g^{HM}/eV	HOMO/eV	LUMO/eV	E_g^{HM}/eV
Z1	-5.6	-3.57	-2.03	-6.51	-2.6	-3.91
Z2	-5.66	-3.65	-2.01	-6.56	-2.67	-3.89
Z3	-5.52	-3.50	-2.02	-6.41	-2.52	-3.89
Z4	-5.49	-3.46	-2.03	-6.39	-2.49	-3.90
Z5	-5.42	-3.38	-2.04	-6.32	-2.4	-3.92

Table S4. Ionization potential (IP), electron affinity (EA) and fundamental gap (E_g^{fund}) calculated by the two functionals.

	B3LYP-D3			ωB97XD		
	IP/eV	EA/eV	E_g^{fund}/eV	IP/eV	EA/eV	E_g^{fund}/eV
Z1	6.44	3.10	3.34	6.51	2.75	3.76
Z2	6.48	3.14	3.34	6.55	2.79	3.76
Z3	6.36	2.98	3.38	6.42	2.63	3.79
Z4	6.29	2.98	3.31	6.37	2.64	3.73
Z5	6.23	2.89	3.34	6.31	2.55	3.76

Table S5. The lowest singlet excitation energy (E_{S1}), lowest triplet excitation energy (E_{T1}) and singlet-triplet energy gap (ΔE_{ST}) calculated by the two functionals.

	B3LYP-D3			ωB97XD		
	E_{T1}/eV	E_{S1}/eV	ΔE_{ST}/eV	E_{T1}/eV	E_{S1}/eV	ΔE_{ST}/eV
Z1	1.25	1.85	0.60	1.33	2.08	0.75
Z2	1.23	1.82	0.59	1.31	2.05	0.74
Z3	1.23	1.85	0.62	1.31	2.07	0.76
Z4	1.24	1.83	0.59	1.32	2.05	0.73
Z5	1.25	1.84	0.59	1.33	2.07	0.74

Table S6. The electron–hole pair binding energy (E_b) calculated by the two functionals.

E_b/eV	Z1	Z2	Z3	Z4	Z5
B3LYP-D3	1.49	1.52	1.53	1.48	1.50
ωB97XD	1.68	1.71	1.72	1.68	1.69

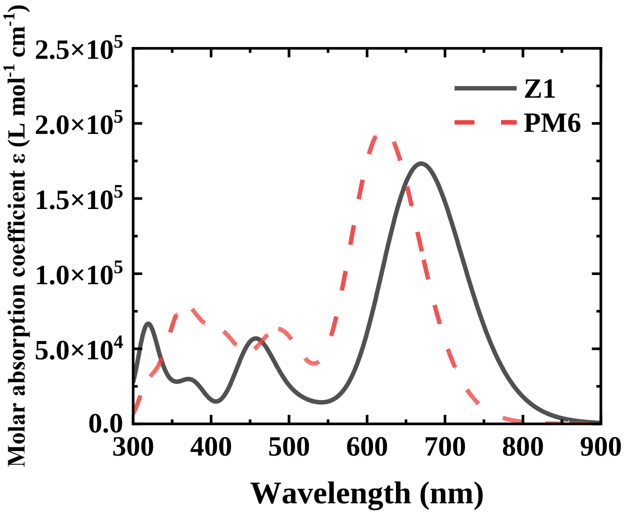

Figure S1. UV-Vis absorption spectra of Z1 and PM6 calculated by B3LYP functional.

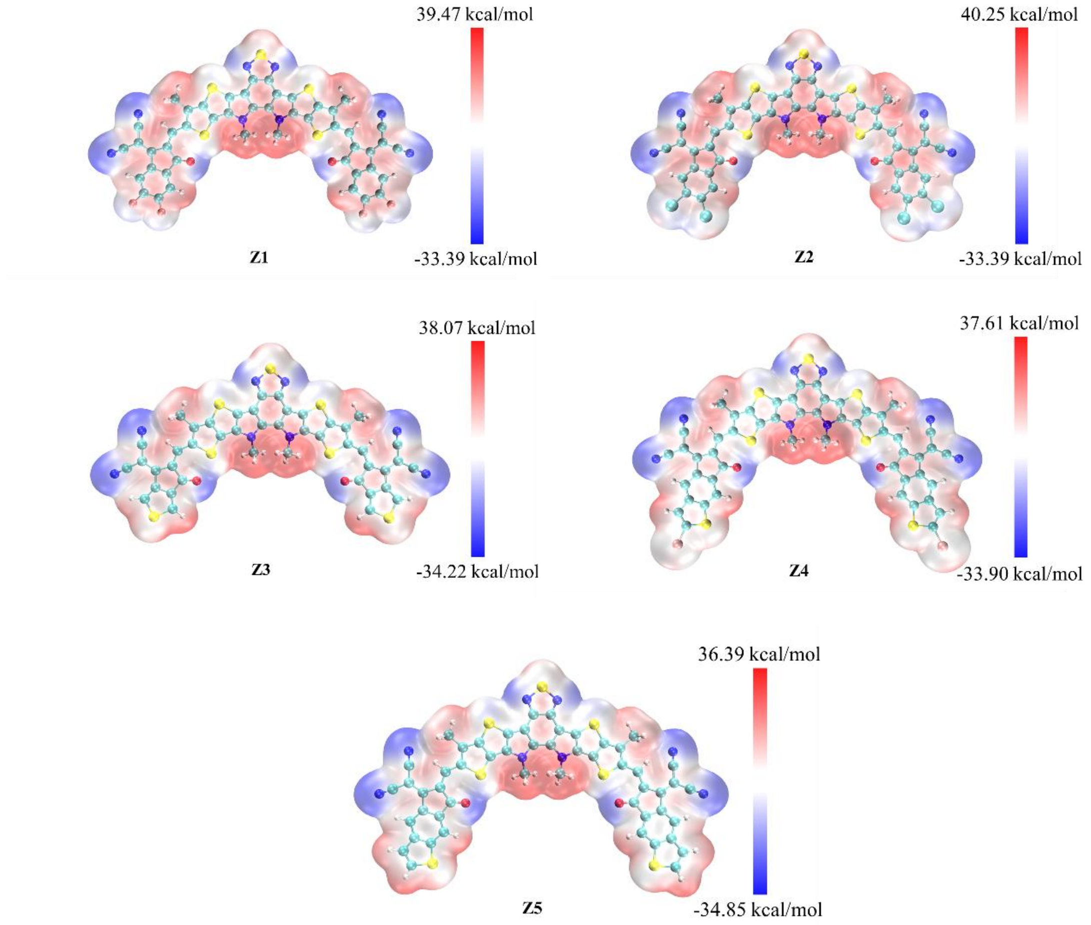

Figure S2. The ESP on the van der Waals surface of the five molecules.

References

[1] C. Li, J. Zhou, J. Song, J. Xu, H. Zhang, X. Zhang, J. Guo, L. Zhu, D. Wei, G. Han, J. Min, Y. Zhang, Z. Xie, Y. Yi, H. Yan, F. Gao, F. Liu, Y. Sun, *Nat. Energy* **2021**, 6, 605.

[2] Y. Cui, H. Yao, J. Zhang, K. Xian, T. Zhang, L. Hong, Y. Wang, Y. Xu, K. Ma, C. An, C. He, Z. Wei, F. Gao, J. Hou, *Adv. Mater.* **2020**, 32, 1908205.
